# A Rare Presentation of T-cell Prolymphocytic Leukemia With Abnormal Uterine Bleeding

**DOI:** 10.14740/jh2152

**Published:** 2026-01-04

**Authors:** Zeinab Nasser, Sarine Tahmazian, Moneb Bughrara, Batoul Nasser, Aula Ramo, Vrushali Dabak

**Affiliations:** a Department of Hematology and Oncology, Henry Ford Hospital, Detroit, MI 48202, USA; b Department of Internal Medicine, Henry Ford Hospital, Detroit, MI 48202, USA; c Department of Internal Medicine, University of Michigan, Ann Arbor, MI 48109, USA; d Department of Radiation Oncology, University of Michigan, Ann Arbor, MI 48109, USA

**Keywords:** T-cell prolymphocytic leukemia, Abnormal uterine bleeding, Lymphadenopathy, Alemtuzumab, Anti-CD52 therapy, Lymphocytosis, Endometrial biopsy

## Abstract

T-cell prolymphocytic leukemia (T-PLL) is a rare and clinically aggressive T-cell neoplasm, which is composed of lymphoid cells that are of post-thymic T-cell origin. This is a case of a 57-year-old female with no significant medical history, who presented with a 4-month history of facial swelling, peripheral edema, dyspnea, palpitations, and abnormal uterine bleeding (AUB). A complete blood count demonstrated lymphocytosis, and a computed tomography (CT) scan of the abdomen and pelvis showed diffuse lymphadenopathy, splenomegaly, and uterine fibroids. A bone marrow biopsy confirmed the presence of T-PLL with a subsequent endometrial biopsy showing atypical lymphoid proliferation consistent with T-PLL. The patient was started on alemtuzumab for T-PLL treatment. This case demonstrates a rare presentation of T-PLL with endometrial involvement, which may have contributed to the patient’s postmenopausal uterine bleeding. Recognition and evaluation of disease infiltration require prompt clinical assessment to reduce the morbidity and mortality associated with T-PLL.

## Introduction

T-cell prolymphocytic leukemia (T-PLL) is an extremely rare form of mature T-cell leukemia that carries a poor clinical outcome due to its rapidly progressing clinical course. T-PLL is composed of lymphoid cells that are of post-thymic T-cell origin and mature phenotype. Clinical presentation includes hepatosplenomegaly, generalized lymphadenopathy, B-symptoms (fatigue, weight loss, night sweats, fevers), excessive lymphocytosis above 100 × 10^9^/L, anemia, and thrombocytopenia. Other commonly seen clinical manifestations include skin, pleural or peritoneal effusions, central nervous system (CNS) involvement, and periorbital, conjunctival, and peripheral edema [1].

T-PLL can be diagnosed by evaluating peripheral blood smear, immunophenotypic markers, and chromosome analysis [1]. Diagnosis is confirmed if three major criteria are met or if the first two major criteria and one minor criterion are met. The three major diagnostic criteria that are required for the diagnosis of T-PLL are a peripheral blood smear or bone marrow biopsy demonstrating cells with the T-PLL phenotype > 5 × 10^9^/L, polymerase chain reaction (PCR) or flow cytometry demonstrating clonality of the T lymphocytes, and expression of T-cell leukemia-1 (*TCL1A/B*) or mature T-cell proliferation *(MTCP1*) genes [1]. Minor diagnostic criteria include abnormalities involving the ataxia telangiectasia mutated (ATM) locus at chromosome 11 (11q22.3), abnormalities in chromosome 8: (8)(p11), t(8;8), trisomy 8q, and abnormalities in chromosomes 5, 12, 13, or 22. It is important to rule out adult T-cell leukemia-lymphoma through serologic and PCR testing for human T lymphotropic virus type 1 (HTLV-1), which should be negative [1]. While it is not necessary to confirm the diagnosis of T-PLL, bone marrow evaluation is required for treatment evaluation and confirmation of remission [1].

Once diagnosis for T-PLL has been established, treatment is indicated for symptomatic patients with active disease. Markers of active T-PLL include patients with constitutional B symptoms, rapidly enlarging lymph nodes, splenomegaly, lymphocytosis, symptomatic bone marrow failure (anemia and/or thrombocytopenia), skin infiltration, pleural effusion, or CNS involvement. Treatment of active T-PLL commonly includes the anti-CD52 monoclonal antibody alemtuzumab, which can achieve complete remission in most patients, though it is not curative. Allogeneic hematopoietic stem cell transplantation is the only known curative treatment for T-PLL. On the other hand, there is no evidence that asymptomatic patients would benefit from treatment; therefore, observation with monthly blood counts and physical examinations for disease progression is recommended. If lymphocyte count doubles within 6 months, this is an indicator of transition into active T-PLL. Despite available therapies, relapse is frequent, highlighting the urgent need for new active treatments to improve the dismal prognosis of T-PLL [2].

## Case Report

### Investigations

A 57-year-old female with no significant past medical history other than known uterine fibroids, presented with a 4-month history of facial edema, bilateral lower extremity edema, abnormal uterine bleeding, shortness of breath, and palpitations. She had a family history of leukemia in her maternal grandmother but was unsure of a specific type. She was a former tobacco smoker, but no other significant psychosocial history. On admission, she was afebrile, and vital signs were stable. Physical exam was significant for pale sclera, abdominal distension, and mild tenderness to abdominal palpation. Neurological and cardiological examinations were normal.

**Figure 1:**
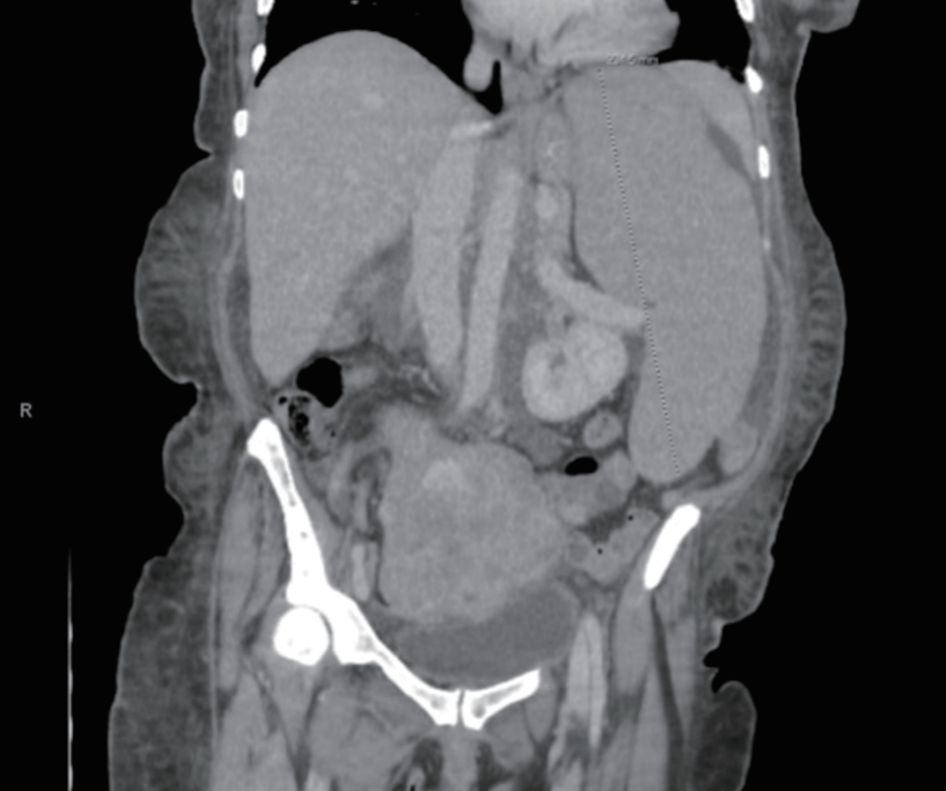
Coronal view of CT chest/abdomen/pelvis showing significant splenomegaly, measuring 23.5 cm in craniocaudal dimension. Enlarged, lobulated uterus with partially calcified and heterogenous fibroids are also seen. CT: computed tomography.

**Figure 2:**
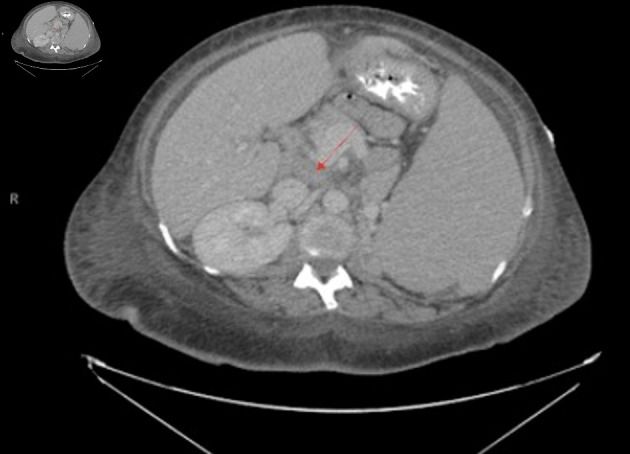
Transverse view of CT chest/abdomen/pelvis showing abdominal lymphadenopathy with noted splenomegaly, with arrow pointing to prominent lymph node. A 3.4 × 2.1 cm nodule on the left adrenal gland is also noted.

Complete blood count (CBC) showed a white blood cell count of 584 × 10^9^/L with absolute lymphocytes of 513 × 10^9^/L, hemoglobin at 2.7 g/dL, mean corpuscular volume (MCV) of 108.7 fL, and platelets at 60 × 10^9^/L. The patient had no previous baseline laboratory studies available for comparison. Iron studies, ferritin, haptoglobin, vitamin B12, and folate were within normal limits. Basic metabolic profile and liver function tests were normal. A peripheral smear was obtained at outside hospital prior to transfer to our hospital, which showed some immature/atypical lymphoid cells, not thought to be blasts, and no presence of Auer rods. Viral studies including HTLV 1/2 antibody, hepatitis, Epstein-Barr virus (EBV), and cytomegalovirus (CMV) were negative. CT scan of the abdomen and pelvis revealed diffuse lymphadenopathy, splenomegaly, and lobulated uterus with partially calcified and heterogenous appearing fibroids (Figs. 1 and 2). The presence of fibroids was confirmed with transabdominal ultrasound, which showed enlarged uterus with multiple masses again most fitting with fibroids. Her uterine contour was irregular, and her myometrium was noted to be heterogenous in texture.

### Diagnosis

**Figure 3:**
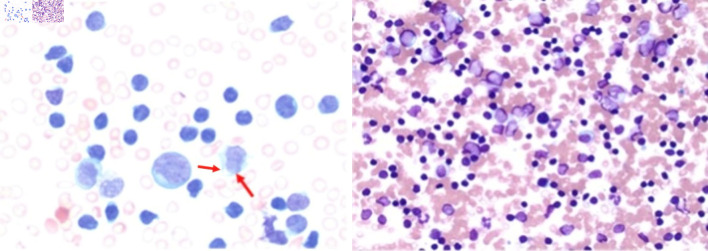
Bone marrow aspirate demonstrating atypical lymphoid cells with an increased nuclear-to-cytoplasmic ratio, irregular nuclear contours, and condensed chromatin (arrows). Cytoplasmic blebs characteristic of leukemic prolymphocytes are present. The bone marrow is nearly completely replaced by atypical lymphoid cells (magnification: × 50 (left) and × 10 (right)).

Obtained bone marrow biopsy was consistent with T-PLL, 90% on the bone marrow aspirate smears and 90% on peripheral blood smear. Bone marrow was hypercellular with markedly diminished trilineage hematopoiesis. Although not pathognomonic, cytoplasmic extensions (blebs) were noted on the peripheral smear (Fig. 3). Flow cytometry on peripheral blood showed a predominant population of CD8+/T-cell receptor (TCR)α/β+ monotypic T cells. No mutations were detected in the *TP53* tumor suppressor gene, and the *BCR-ABL1* fusion gene was negative. Fluorescence *in situ* hybridization (FISH) studies were positive for inv(14) with *TRAD* gene rearrangement.

Due to her irregular and prominent uterine bleeding, an endometrial biopsy was obtained, and pathology was consistent with T-PLL. It found evidence of cells with inv(14) *TRAD* gene (14q11.2). Forty-five percent of cells reviewed showed separation of *TRAD*. Endometrial HPV testing was also negative. These findings demonstrated a unique progression of cancer cells into the endometrial lining.

In review, the presence of inv(14) on FISH is highly specific for T-PLL. This genetic feature helped delineate T-PLL from other disorders with similar manifestations such as peripheral T-cell lymphoma and indolent T-cell lymphoproliferative disorders. Alterations in *ATM* gene, which are also highly specific for T-PLL, were not evaluated.

### Treatment

The patient was initiated on alemtuzumab, an anti-CD52 monoclonal antibody, for treatment of T-PLL and completed the induction phase and first cycle of therapy, consisting of 11 sessions. She received one treatment of cycle two, then therapy was stopped. Her treatment schedule was interrupted multiple times due to failure to attend scheduled appointments and frequent hospitalizations for coronavirus disease 2019 (COVID-19) and dyspnea evaluations.

### Follow-up and outcomes

Unfortunately, with progression of her disease, she experienced recurrent hospitalizations for pneumonia and worsening generalized anasarca. Four months after diagnosis, a joint decision was made by the patient and family to pursue home hospice care.

## Discussion

T-PLL is a rare and aggressive mature T-cell neoplasm of lymphoid cells encompassing 2% of mature lymphocytic leukemias in middle-aged adults [1]. The incidence of T-PLL is extremely low, occurring only in 0.6 cases per million individuals [2]. Because multiple leukemias can present with similar clinical features, differentiation of T-PLL may be difficult. It is important to differentiate T-PLL from other entities such as Sezary syndrome, B-cell prolymphocytic leukemia (B-PLL), adult T-cell lymphoma/leukemia (ATLL), and chronic lymphocytic leukemia (CLL). Although B-PLL can present similarly to T-PLL, it generally lacks skin involvement and lymphadenopathy, which are more characteristic of Sezary syndrome and T-PLL. Therefore, a skin biopsy would be essential to rule out Sezary syndrome. ATLL typically presents with HTLV-1 positivity, but our patient was negative for HTLV-1 [3].

Peripheral blood smear can aid in diagnosis but is often nonspecific. On blood smear, T-PLL morphology has three main variants: small to medium-sized cells, small-sized cells, and the “cerebriform Sezary-like” variant, which defines an irregular nuclear outline [3]. The most common variant, composed of small- to medium-sized lymphoid cells, exhibits irregular, round, or oval nuclei with basophilic cytoplasm-like granules. T-PLL commonly demonstrates cytoplasmic protrusions, referred to as blebs, a morphologic feature that aids in distinguishing it from other mature T-cell leukemias [3]. Due to the presence of a cerebriform variant that presents morphologically like Sezary, blood smear alone would not differentiate Sezary from T-PLL. CLL can be differentiated from T-PLL due to the presence of “smudge cells” in CLL on blood smear [4]. Ultimately, the diagnosis of T-PLL cannot be made with blood smear alone, with cytological analysis and immunophenotyping being the best tools to allow for differentiation amongst the other various leukemias. T-PLL tends to be positive for CD2, CD3, CD5, CD7, and CD52. The presence of gene rearrangements in either *TCL1A*, *TCL1B*, or *MTCP1* are very specific for T-PLL [1].

Although thrombocytopenia is a common finding in patients with T-PLL, to our knowledge no other cases have presented with abnormal uterine bleeding. Furthermore, only one other case has demonstrated involvement of the uterus, which was discovered on postmortem analysis. The patient, a 34-year-old female, was diagnosed with T-PLL 13 months after a splenectomy, following her death. Autopsy revealed extensive leukemic infiltration affecting several organs, including the uterus and adrenal glands [5]. The most common sites of T-PLL involvement include lymph nodes, liver, spleen, and skin [3]. In this case, uterine involvement might not have been detected had an endometrial biopsy not been performed for evaluation of abnormal uterine bleeding. Furthermore, given that abnormal uterine bleeding was a prominent presenting symptom in our patient, the unique uterine involvement of T-PLL might have gone unrecognized had the initial CBC not prompted a hematologic evaluation. Her marked anemia was more likely attributable to leukemic bone marrow infiltration and replacement rather than acute blood loss from uterine bleeding. The proper workup was essential to help establish the diagnosis of T-PLL over a malignancy of uterine origin. There are primary and secondary lymphomas of the genital tract including diffuse large B-cell lymphoma, extra nodal marginal zone lymphoma, peripheral T-cell lymphoma, and adult T-cell leukemia. The presence of T-PLL within the endometrium was established by FISH analysis, which demonstrated a positive inversion of chromosome 14 involving the *TRAD* gene at 14q11.2. This finding highlights the critical role of cytogenetic and immunohistochemical analyses in elucidating the molecular mechanisms underlying cellular dysregulation in hematologic malignancies.

Urgent diagnosis, as in our case, is essential due to the mortality associated with T-PLL. Untreated T-PLL has been shown to have a mortality rate less than 1 year [6]. Even with alemtuzumab therapy and subsequent allogeneic hematopoietic stem cell transplantation, median survival is limited to approximately 1 - 3 years, and few patients achieve complete remission [2]. Alemtuzumab has been shown to be an effective treatment for resolution of symptoms in our patient’s case as well as for others [2]. Nevertheless, the disease remains highly lethal, with many patients ultimately dying from disease-related complications. Alemtuzumab is used off-label and is not Food and Drug Administration (FDA)-approved for the treatment of T-PLL. In addition, with only a fraction of patients with T-PLL eligible for allogeneic hematopoietic stem cell transplant, there is a great need for further treatment options and development. With a better understanding of the vulnerabilities of the disease, greater potential treatment options targeting various molecular and genetic pathways may come to fruition [7].

### Learning points

This case serves to demonstrate the clinical suspicion and thorough workup needed to diagnose T-PLL and differentiate it from other leukemias. Diagnosis may be difficult with many nonspecific symptoms including constitutional symptoms, facial/peripheral edema, and possible atypical symptoms due to rare organ involvement such as abnormal uterine bleeding. While T-PLL presents with evidence of bone marrow failure, it is crucial to recognize other organ involvement including lungs, spleen, liver, skin and in this case, the uterus. Awareness of this pattern of involvement highlights the need for careful integration of clinical symptoms and objective data to guide diagnostic evaluation and prompt treatment initiation. Cytological analysis and immunophenotyping are the most vital tools in developing and confirming a diagnosis of T-PLL. Although patients with active T-PLL are candidates for off-label alemtuzumab therapy and allogeneic hematopoietic bone marrow transplant, there is a greater need to develop additional treatment options.

## Data Availability

The authors declare that data supporting the findings of this study are available within the article.

## Abbreviations

T-PLL: T-cell prolymphocytic leukemia
TCL1A/B: T-cell leukemia-1
CBC: complete blood count
MCV: mean corpuscular volume
MTCP1: mature T-cell proliferation
B-PLL: B-cell prolymphocytic leukemia
ATLL: adult T-cell lymphoma/leukemia
CLL: chronic lymphocytic leukemia
AUB: abnormal uterine bleeding
